# Integrated analysis of fibroblasts molecular features in papillary thyroid cancer combining single-cell and bulk RNA sequencing technology

**DOI:** 10.3389/fendo.2022.1019072

**Published:** 2022-10-26

**Authors:** Wei Li, Zhiyong Liu, Xiaoxia Cen, Jing Xu, Suo Zhao, Bin Wang, Wei Zhang, Ming Qiu

**Affiliations:** ^1^ Department of General Surgery, Changzheng Hospital, Navy Medical University, Shanghai, China; ^2^ Department of Gastroenterology and Hepatology, Zhongshan Hospital, Fudan University, Shanghai, China

**Keywords:** thyroid cancer, single-cell sequencing, tumor environment, fibroblasts, prognosis

## Abstract

**Background:**

Papillary thyroid cancer (PTC) is the most common pathological type of thyroid cancer with a high incidence globally. Increasing evidence reported that fibroblasts infiltration in cancer was correlated with prognostic outcomes. However, fibroblasts related study in thyroid cancer remains deficient.

**Methods:**

Single-cell sequencing data of PTC were analyzed by Seurat R package to explore the ecosystem in PTC and identify fibroblasts cluster. The expression profiles and prognostic values of fibroblast related genes were assessed in TCGA dataset. A fibrosis score model was established for prognosis prediction in thyroid cancer patients. Differentially expressed genes and functional enrichment between high and low fibrosis score groups in TCGA dataset were screened. The correlation of immune cells infiltration and fibrosis score in thyroid cancer patients was explored. Expression levels and prognostic values of key fibroblast related factor were validated in clinical tissues another PTC cohort.

**Results:**

Fibroblasts were highly infiltrated in PTC and could interact with other type of cells by single-cell data analysis. 34 fibroblast related terms were differentially expressed in thyroid tumor tissues. COX regression analysis suggested that the constructed fibrosis score model was an independent prognostic predictor for thyroid cancer patients (HR = 5.17, 95%CI 2.31-11.56, P = 6.36E-05). Patients with low fibrosis scores were associated with a significantly better overall survival (OS) than those with high fibrosis scores in TCGA dataset (P = 7.659E-04). Specific immune cells infiltration levels were positively correlated with fibrosis score, including monocytes, M1 macrophages and eosinophils.

**Conclusion:**

Our research demonstrated a comprehensive horizon of fibroblasts features in thyroid cancer microenvironment, which may provide potential value for thyroid cancer treatment.

## Introduction

Thyroid cancer remains to be the most common endocrine malignant tumor globally (1). It is estimated that 3% females in the United states were newly diagnosed with thyroid cancer in 2021 (2). Papillary thyroid cancer (PTC) is the most common pathological type of the disease. Though mortality of PTC is comparatively low, 5-30% of patients with PTC suffer from recurrence (3, 4). The risk stratification system proposed by the American Thyroid Association (ATA) integrates the perioperative clinicopathological data, which mainly provides prognostic information (5). It’s difficult to select individualized therapy based on the traditional risk stratification system to avoid over-treatment. and thus, a molecular diagnosis of suspicious nodules is more recommended for decision-making before intervention (6, 7). Approximately 45% of PTC patients are detected with BRAF-V600E mutation, which is associated with poor prognosis (8). However, the role of BRAF inhibitors in non-BRAF mutated cancers is reported to be controversial (9). Consequently, a combined molecular diagnostic test is necessary for decision making in the future.

The tumor microenvironment (TME), consisting of stroma cells, immune cells, chemokines as well as extracellular matrix (ECM), has been shown to play significant roles in prognosis of disease (10, 11). Immune cells were reported to play a critical pro- or anti-tumor role in PTC progression, such as macrophages, MDSC (myeloid-derived suppressor cells), neutrophils, Tregs (regulatory T cells), dendritic cells (DCs) (12, 13). Fibroblasts are one of the significant stroma cells in TME. The interactive relationship of fibroblasts and immune cells during PTC development were largely unclear. In cancer stroma, resident fibroblasts could be activated by transforming growth factor-beta (TGF-beta) and develop into cancer-associated fibroblasts (CAFs) (14). Moreover, a variety of distant cells, consisting of epithelial cells, bone marrow mesenchymal stem cells (BM-MSCs) and adipocytes, could differentiated into CAFs and be recruited to tumor sites (15). Generally, CAFs have been reported to promote cell proliferation, invasion, as well as angiogenesis and induce chemoresistance in several cancers, such as colorectal cancer (16), breast cancer (17) and pancreatic cancer (18). Recent studies have revealed that CAF is associated with dedifferentiation, invasion, and lymph nodes metastasis of thyroid cancer (19–21). However, the molecular mechanism of CAFs in PTC is still under discussed.

The purpose of this study was to systematically explore the microenvironment in PTC development and reveal the expression features of fibroblasts through single-cell and bulk RNA sequencing technology. We found that fibroblasts were highly infiltrated in PTC when compared with normal thyroid tissues, and could widely interact with immune cells. By analyzing the fibroblast related genes, we constructed a novel fibrosis score model for PTC patients, which indicated satisfactory survival prediction. The differentially expressed genes (DEGs) and immune cell infiltration changes between high and low fibrosis score groups were also explored. Our research provided systematically insights about fibroblasts features, which might serve as critical targets in PTC treatment.

## Materials and methods

### Data acquisition

The papillary thyroid carcinoma (PTC) single-cell RNA sequencing data of GSE184362 (22) and bulk transcriptional sequencing data of GSE33630 (23) were downloaded from the Gene Expression Omnibus (GEO) (https://www.ncbi.nlm.nih.gov/geo/). Clinical information and RNA sequencing data of thyroid cancer (THCA) patients were obtained from The Cancer Genome Atlas (TCGA) database (https://portal.gdc.cancer.gov/).

### Single-cell data processing and cell cluster identification

The single-cell sequencing data of GSE184362 were downloaded from portal website and 3 tumor and 3 para-tumor tissues were analyzed by Seurat R (4.0.2) package. The low-quality cells were filtered with the criteria: mitochondrial genes more than 8%, nFeature RNA less than 200 and more than 4000. Then we scaled the data and used the “RunPCA” function for dimension reduction. The “FindNeighbors” and “FindClusters” functions with the resolution of 0.5 in Seurat were used for cell clustering. A t-distributed stochastic neighbor embedding (t-SNE) was applied to visualize single-cell clustering. To identify the cell clusters, the differently expressed genes of each cell clusters were screened with the criteria of log2FC > 0.25 using “FindAllMarkers” function in Seurat. The marker genes of each cell type were referenced as previous literatures and CellMarker database (http://bio-bigdata.hrbmu.edu.cn/CellMarker/).

### Cell-cell communication

The CellChat (1.1.3) R package was performed to explore the communications and the interacting molecules mechanisms between the 11 cell clusters in thyroid cancer, including CD8 T cells, CD4 T cells, B cells, Treg cells, plasma cells, natural killer (NK) cells, myeloid cells, monocytes, thyrocytes, fibroblasts, and endothelial cells. The communication network was analyzed and visualized with the “aggregateNet” function in CellChat package based on the interacting counts and weight between cell clusters. The incoming and outgoing signaling roles of the aggregated cell-cell communication network were explored by the function “netAnalysis_signalingRole_heatmap” in CellChat package.

### Derivation and analysis of fibroblasts gene list in thyroid cancer

Based on the single-cell analysis data, the specific gene list in fibroblasts cluster was selected using the criteria log2FC>2 and P value<0.05. A total of 100 terms were identified as fibroblast related genes (FRGs). To compare the expression levels of FRGs in thyroid carcinoma and normal tissues, the TCGA-TPCA dataset was explored using limma and edgeR packages in R. The differentially expressed FRGs (DEFRGs) between thyroid tumor and normal tissues were visualized by heatmap R package. To analyze the correlations of DEFRGs, corrplot R package was applied by Pearson correlation analysis. We further divided the THCA patients into early-stage (I and II) and advanced stage (III and IV) groups, and compared the expression of DEFRGs between two groups.

### Fibrosis score model construction in thca patients

Univariate Cox regression analysis was conducted to evaluate the independent prognostic values of DEFRGs. We further performed least absolute shrinkage and selection operator (LASSO) regression analysis with glmnet R package to pick the critical prognosis related DEFRGs and calculate the regression coefficient. Six DEFRGs (PCOLCE2, APOD, APOE, TIMP1, HTRA3 and MT1A) were selected to evaluate the fibrosis score of each patient in TCGA-THCA dataset. The formula for fibrosis score calculation as follow: fibrosis score = 0.236*PCOLCE2 + 0.033*APOD - 0.274*APOE - 0.065*TIMP1 + 0.237*HTRA3 - 0.065*MT1A. Receiver operating characteristic (ROC) curve analysis was applied to validate the prognostic signature of fibrosis score. Subsequently, patients with THCA were divided into high fibrosis score and low fibrosis score groups based on the median fibrosis value. The overall survival rates between two groups were analyzed using Kaplan−Meier survival analysis with log−rank test. The nomogram plot model was constructed to predict the prognostic outcomes of THCA patients based on the fibrosis scores and clinical features using survival and rms packages in R. The calibration curves of 1-year, 5-year and 10-year survival proportion were plotted to assess the predicting efficiency of nomogram.

### Cox regression analysis

Univariate and multivariate Cox regression analysis were performed with coxph function in survival R package to evaluate the prognostic values of fibrosis score and clinical features of patients, including age, gender, cancer type, BRAF mutation and stage.

### Correlation analysis between fibrosis score and cancer associated fibroblast (CAF) marker genes

The myofibroblastic CAF (myoCAF) and inflammatory CAF (iCAF) are two main types of CAFs. According to the previous researches, 10 markers of myoCAF (ACTA2, TAGLN, MMP11, MYL9, HOPX, POSTN, TPM1, TPM2, IGFBP7, and CST1) and 10 markers of iCAF (PLA2G2A, CCDC80, MCL1, S100A10, LMNA, UAP1, DPT, ABL2, EFEMP1 and TNFAIP6) were selected in TCGA-THCA dataset. The correlation between the expression of myoCAF and iCAF marker genes and fibrosis score were analyzed using corrplot R package.

### DEGs analysis and functional enrichment between two fibrosis score groups

Limma and edgeR packages of R were applied to investigate the transcriptional difference of THCA patients between high and low fibrosis score groups. The DEGs were selected with an absolute Log2 (FC) value>0.5 and p value<0.05. Expression of DEGs and other clinical information were visualized using heatmap R package. Gene ontology (GO) terms and Kyoto Encyclopedia of Genes and Genomes (KEGG) pathway enrichment analysis were performed with The R clusterProfiler package.

### Immune cell infiltration analysis

CIBERSORT R package containing 22 gene sets of immune cell types was applied to evaluate the infiltrating abundance of each type of immune cells in THCA patients. Infiltration levels of immune cells were further compared between high and low fibrosis score groups. The correlation between immune cell infiltration and fibrosis score was investigated using Pearson correlation analysis in GraphPad Prism 9.0. The ESTIMATE algorithm was used to quantify the immune and stromal score in each tumor sample. Recent evidence identified that a 12 chemokines signature (CCL2, CCL3, CCL4, CCL5, CCL8, CCL18, CCL19, CCL21, CXCL9, CXCL10, CXCL11, and CXCL13) was highly correlated with tertiary lymphoid structures (TLS). As previously reported, we calculated TLS score according the level of 12 genes of each sample using ssGSEA method by R-package (24, 25).

### Clinical samples

PTC tissues and normal thyroid tissues were obtained from 20 surgery PTC patients with informed consent at the Department of General Surgery, Changzheng Hospital (Shanghai, China). All clinical tissues were immediately frozen in liquid nitrogen after surgery and stored at – 80 °C. This study was approved by the Research and Ethics Committee of Changzheng Hospital. The clinical information of PTC patients was listed in **Table S3**.

### RNA extraction and RT-qPCR

Total RNA of clinical tissues was isolated Trizol (Invitrogen, USA) according to the manufacturer’s protocols. The concentration isolated RNA was analyzed by NanoDrop1000 (ThermoFisher, USA). The purified RNA was reverse-transcribed using Hifair^®^ II 1st Strand cDNA Synthesis Kit (Yeasen, China). The cDNA was used for qPCR analysis using Hieff UNICON^®^ qPCR SYBR Green Master Mix (Yeasen, China). The expression of β-actin was applied as endogenous control. The primers for qPCR are shown in **Table S4**.

### Statistics

All analyses in this study were performed with R software 4.0.3 and GraphPad Prism 9.0. The t-test and Mann-Whitney U test were applied for comparisons between two groups. Kaplan–Meier method with log-rank test was used for survival analysis. Time-dependent ROC curves were applied to assess the accuracy of the fibrosis score model. Univariate and multivariate Cox regression analysis were used to determine the independent prognostic values of fibrosis score and key FRGs. The Pearson correlation analysis was used to analyze the correlation coefficient between two variables. P value < 0.05 was considered statistically significant.

## Results

### Single-cell atlas of thyroid cancer patients

**Figure 1:**
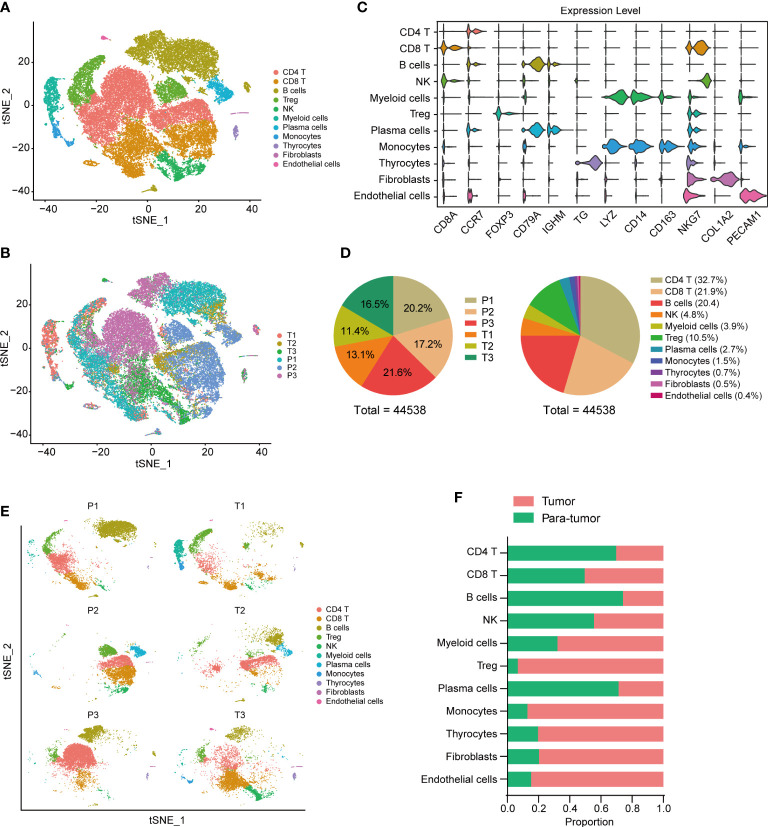
Tumor environment in thyroid cancer. t-SNE visualization of tumor ecosystem in thyroid cancer. Colored by cell clusters **(A)** and sample tissues **(B)**. **(C)** Violin plots of cell markers in 11 clusters. **(D** Cell proportions in each sample and cell cluster. **(E)** t-SNE visualization of cell clusters in each sample. **(F)** Proportions of different cell clusters in tumor and para-tumor thyroid tissues.

To investigate the tumor heterogeneity of thyroid cancer, we analyzed the single-cell data of 6 thyroid tissues, including 3 tumor tissues and 3 para-tumor tissues. After filtering low-quality data and reducing the dimension, we identified a total of 44538 cells and 11 cell clusters, including CD4 T cells, CD8 T cells, B cells, Treg cells, plasma cells, natural killer (NK) cells, myeloid cells, monocytes, thyrocytes, fibroblasts, and endothelial cells in 6 samples (**Figures 1A, B**). CD4 T, CD8 T and Treg cells were labeled with the markers CD8A, CCR7, CTLA4 and FOXP3. B cells and plasma cells were labeled with CD79A, CD79B, MS4A1 and IGHM. NK cells were labeled with NKG7, KLRD1, KLRF1 and FCGR3A. Myeloid cells and monocytes were labeled with LYZ, S100A8, S100A9, CD14, CD163, CD68, HLA−DPA1 and HLA−DRB5. Thyrocytes were labeled with TG, TPO, EPCAM and KRT18. Fibroblasts were labeled with COL1A1, COL1A2, COL3A1 and ACTA2. Endothelial cells were labeled with PECAM1, CD34, CDH5 and VWF. The expression dot plot and violin plot of marker genes were shown in **Figures 1C**, **S1A**. The cell proportions of each sample and cell cluster were presented at **Figure 1D**. We further explored the 11 cell clusters in each sample and compared cell proportions between tumor and para-tumor tissues (**Figures 1E, F**, **S1B**). We found relative low proportions of CD4 T cells, B cells and plasma cells in tumor tissues, while high proportions of Treg, monocytes, thyrocytes, fibroblasts, and endothelial cells were seen in tumor tissues (**Figure 1F**).

### Cell-cell communication analysis in thyroid cancer

**Figure 2:**
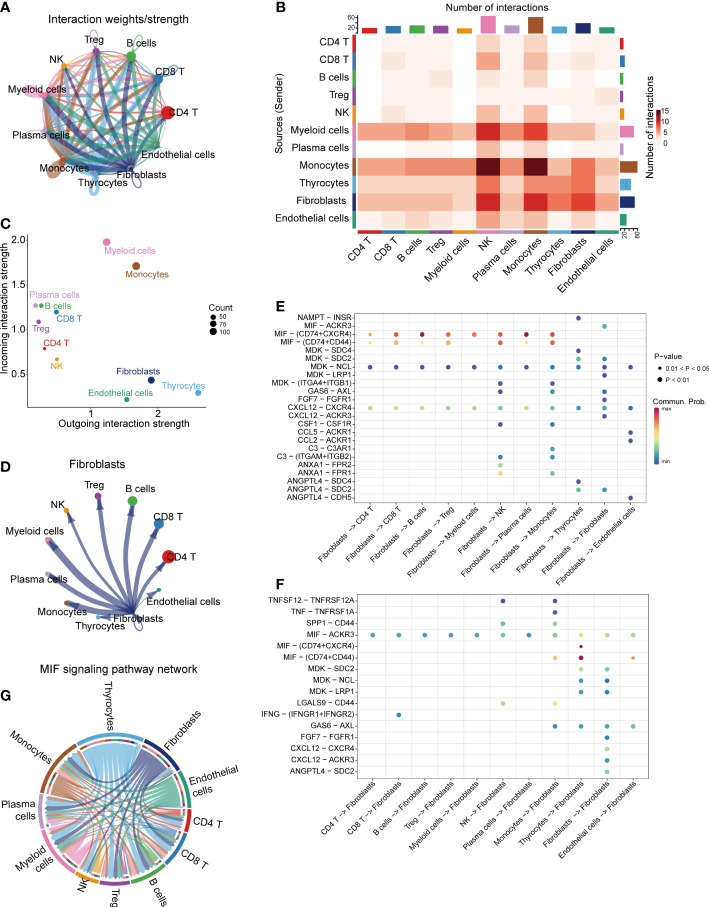
Cell-cell communications in thyroid cancer. **(A)** Integrated cell-cell communications network plotted by interaction weights. **(B)** The cell-cell communications heatmap. **(C)** The dot plot of outgoing/incoming interaction strength for 11 cell clusters. **(D)** Cell-cell communications network of fibroblasts. The dot plot of fibroblasts outgoing **(E)** and incoming **(F)** interaction signal pathways. **(G)** MIF signaling pathway network.

CellChat R package was performed to explore the cell-cell communications and delineate the interacting signal pathways in single-cell data. The aggregated cell-cell communication networks were constructed by interaction weights (**Figure 2A**) and interaction numbers (**Figure S1C**). The interaction heatmap between each cell cluster was shown in **Figure 2B**, indicating the significant interacting strengths of different cell types. The cell outgoing and incoming interaction strengths were plotted at **Figure 2C**, which suggests that myeloid cells mostly receive messages from other cells, while thyrocytes and fibroblasts play critical roles as message sender. The separate communication network showed that fibroblasts had widespread interactions with other cell types (**Figures 2D**, **S1D**). To further clarify the potential cell-cell communications between fibroblasts and other cell types, we analyzed the different outgoing and incoming signal pathways of fibroblasts based on the relative expression of ligand-receptor (L-R) pairs (**Figures 2E, F**, **S2A**). We found that fibroblasts could significantly interact with most of immune cells *via* CD74-CXCR4 and CD74-CD44 complexes in macrophage migration inhibitory factor (MIF) signal pathway, CXCL12−CXCR4 interactions and MDK−NCL interactions (**Figure 2E**), while fibroblasts received interactions from other cells *via* MIF-AKCR3 pathway (**Figure 2F**). The MIF signal pathway communication networks between cell clusters were selected and shown in **Figures 2G**, **S2B**.

### Fibroblast related genes (FRGs) analysis and fibrosis model establishment

**Figure 3:**
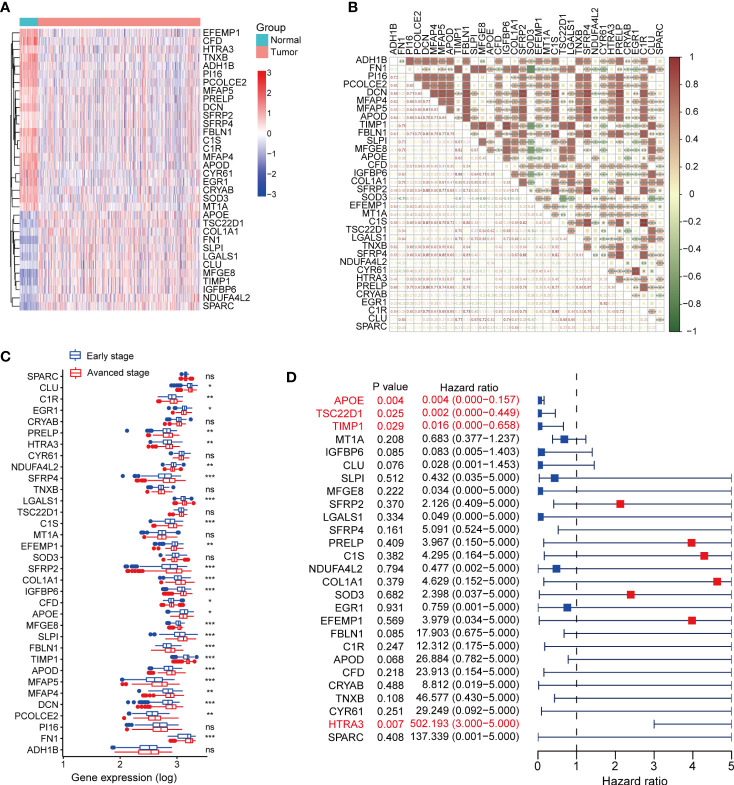
Expression profiles of fibroblast related genes in TCGA-THCA dataset. **(A)** The expression heatmap of DEFRGs between normal and tumor thyroid cancer. **(B)** The correlation between DEFRGs. **(C)** Comparing the expression of DEFRGs in early stage and advanced stage of thyroid cancer. **(D)** Univariate Cox regression analysis the prognostic values of DEFRGs. *P < 0.05; **P < 0.01; ***P < 0.001.

We further analyzed the expression set of fibroblasts in single-cell data and identified 100 fibroblast related genes (FRGs) with the criteria log2FC>2 and P value<0.05 (**Table S1**). The expression of FRGs was explored in TCGA-THCA dataset (**Figure S3A**), and 34 terms were differentially expressed between tumor and normal thyroid tissues, including 12 up-regulated genes and 22 down-regulated genes in thyroid cancer. The heatmap of 34 DEFRGs was plot in **Figure 3A**. We further analyzed the correlations between DEFRGs and found that most of DEFRGs were significantly positively correlated with others (**Figure 3B**). To explore the association between the expression of the 34 DEFRGs and clinical stages, we divided the patients into early-stage group, which contains stage I and II patients, and advanced stage group, containing stage III and IV patients. We found the expression levels of 25 DEFRGs were associated with clinical stage (**Figure 3C**). Univariate Cox regression analysis was further applied to assess the prognostic value of DEFRGs, and we found that 4 DEFRGs (APOE, P = 0.004; TSC22D1, P = 0.025; TIMP1, P = 0.029; HTRA3, P = 0.007) may act as independent factors for overall survival of thyroid cancer (**Figure 3D**).

**Figure 4:**
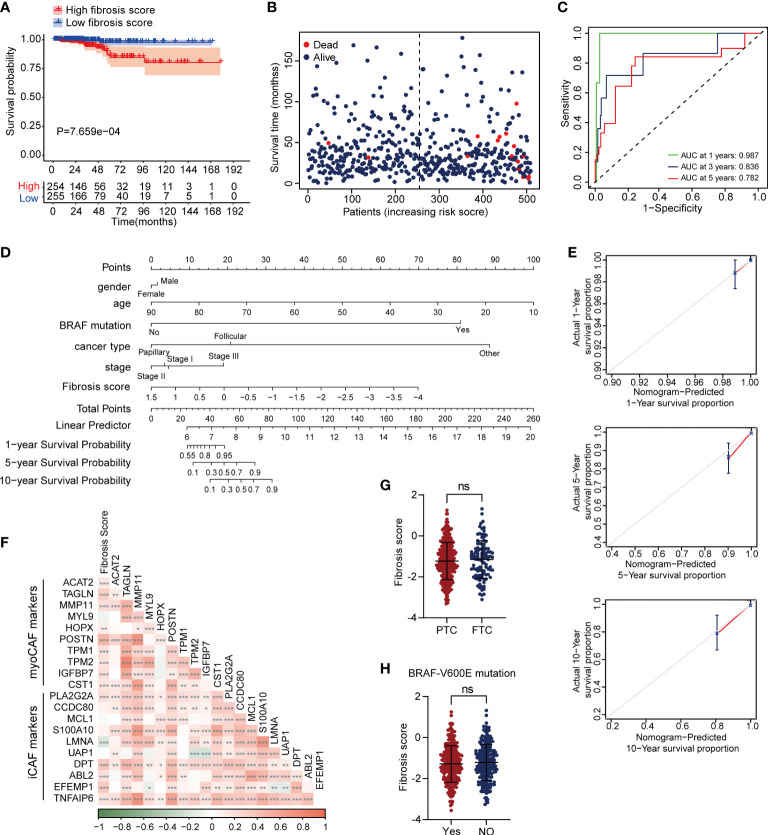
High fibrosis score was associated with poor overall survival in thyroid cancer patients. **(A)** Kaplan–Meier plot of fibrosis scores showed high fibrosis score patients had poor prognostic outcomes. **(B)** The distribution of fibrosis scores and survival status of cases in TCGA-THCA dataset. **(C)** The time dependent ROC plot of fibrosis score. **(D)** A nomogram plot for survival predication in thyroid cancer patients. **(E)** The calibration curve of nomogram showed optimal prognosis predicating efficiency. **(F)** The correlation between fibrosis score and marker genes of myoCAF and iCAF. Fibrosis score showed no significant difference between PTC and follictalar thyroid carcinoma (FTC) **(G)**, as well as BRAF-V600E mutation **(H)**. *P < 0.05; **P < 0.01; ***P < 0.001.

We performed LASSO regression analysis and selected 6 critical prognostic DEFRGs (PCOLCE2, APOD, APOE, TIMP1, HTRA3 and MT1A). Based on the expression value and regression coefficient of 6 DEFRGs, we calculated fibrosis score for each case in TCGA-THCA dataset, and further divided the patients into high fibrosis score group and low fibrosis score group by median value of fibrosis score (**Figure S3B**). Survival curves indicated that high fibrosis score group patients were associated with relatively poor overall survival (OS) outcomes in TCGA-THCA dataset (**Figure 4A**). The distribution of fibrosis scores and prognostic status of patients were shown in **Figure 4B**. The receiver operating curve (ROC) suggested that fibrosis scores displayed powerful efficacy for predicting the overall survival (OS) probability, as the area under the curve (AUC) of 1-year, 3-year, and 5-year OS probability were 0.987, 0.836, and 0.782, respectively (**Figure 4C**). By performing univariate and multivariate Cox analysis, we found that the fibrosis score (HR = 5.17, 95%CI 2.31-11.56, P = 6.36E-05) along with stage (HR = 3.75, 95%CI 1.19-11.79, P =0.024) might serve as critical factors in predicting thyroid cancer prognosis (**Table 1**). We further constructed a nomogram combining the clinical characteristics and fibrosis score to assess the survival status for patients in TCGA-THCA (**Figure 4D**). The calibration curve indicated that the nomogram exhibited optimal predicating performance (**Figure 4E**). As pervious reports, cancer associated fibroblasts (CAFs) had two main types, myofibroblastic CAF (myoCAF) and inflammatory CAF (iCAF). To explore the association between different types of CAFs and fibrosis score, we selected 10 marker genes expressed in myoCAF and iCAF. Pearson correlation analysis showed that fibrosis score was positively correlated with most of markers, which negatively correlated with LMNA expression, a marker gene of iCAF (**Figure 4F**). No significant difference of fibrosis score was found between different thyroid cancer types and BRAF mutation states (**Figures 4G, H**).

**Table 1:** Univariate and multivariate Cox regression analyses of fibrosis score in TCGA-THCA database.

Variables	Univariate Cox		Multivariate Cox	
	HR (95%CI)	P value	HR (95%CI)	P value
Age (≥ 55 vs. < 55)	2.67E+09 (0-inf)	0.99	–	–
Gender (female vs. male)	0.51 (0.18-1.41)	0.19	–	–
Tumor type (others vs. PTC)	0.29 (0.04-2.23)	0.24	–	–
BRAF mutation (yes vs. no)	1.09E-07 (0-inf)	0.99		
Stage (III + IV vs. I + II)	7.26 (2.34-22.57)	6.11E-04	3.75 (1.19-11.79)	0.024
Fibrosis score	5.93 (2.84-12.38)	2.14E-06	5.17 (2.31-11.56)	6.36E-05

### DEGs analysis and signaling pathways enrichment between high- and low- fibrosis score groups

**Figure 5:**
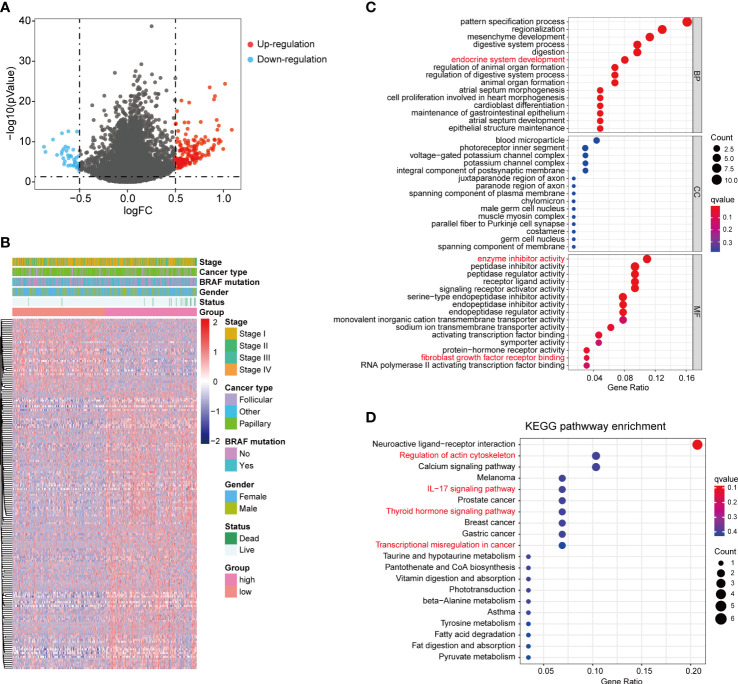
DEGs between high and low fibrosis score patients. **(A)** The volcano plot of DEGs between high and low fibrosis score patients. **(B)** The expression heatmap of DEGs and clinical features in thyroid cancer patients. **(C)** GO enrichment of DEGs. **(D)** KEGG enrichment of DEGs.

To investigate the potential biological differences of the two fibrosis score groups, we compared the transcriptional expression between two groups and screened 186 DEGs, which included 147 up-regulated genes and 39 down-regulated genes in high fibrosis score group (**Figures 5A, B**). The fold change and mean expression of DEGs in two groups were listed in **Table S2**. The GO terms were identified based on the DEGs, and the top 15 biological process (BP), cellular component (CC) and molecular function (MF) were showed in bubble plot (**Figure 5C**). We detected several interested GO terms, such as “endocrine system development”, “enzyme inhibitor activity” and “fibroblast growth factor receptor binding”. The top 20 KEGG signal pathways were presented in **Figure 5D**, which involved in “regulation of actin cytoskeleton”, “IL−17 signaling pathway”, “thyroid hormone signaling pathway” and “transcriptional mis-regulation in cancer”.

### High fibrosis score was associated with immune cell infiltration

**Figure 6:**
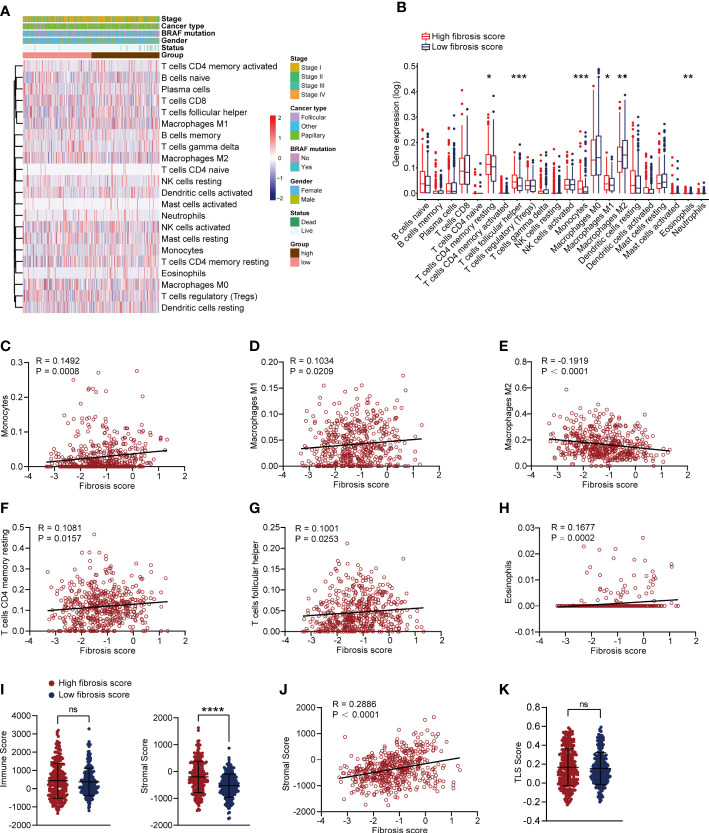
Correlation analysis of immune cells and fibrosis scores in thyroid cancer patients. **(A)** The heatmap of immune cells infiltration and clinical features in thyroid cancer patients. **(B)** Comparing the infiltration levels of immune cells between high and low fibrosis score patients. **(C–H)** Correlation analysis between fibrosis scores and monocytes, M1 macrophages, M2 macrophages, CD4 memory resting T cells, follicular helper T cells and eosinophils. **(I)** Stromal scores were increased in high fibrosis score group, but no significant difference was found in immune scores. **(J)** Stromal scores were positively correlated with fibrosis scores in thyroid cancer patients. **(K)** No significant difference of TLS score was found between high and low fibrosis score groups. *P < 0.05; **P < 0.01; ***P < 0.001.

We assessed the immune cell infiltration levels in each patient from TCGA-THCA dataset using CIBERSORT algorithm, and the results were shown in **Figure S4A**. The heatmap of immune cell infiltration in two fibrosis score groups were presented in **Figure 6A**. We further compared the infiltration changes of immune cells between the two groups (**Figure 6B**) and found that CD4 memory resting T cells, follicular helper T cells, monocytes, M1 macrophages and eosinophils exhibited high infiltration levels, while M2 macrophages showed attenuated infiltration level in high fibrosis score group. Correlation analysis revealed that fibrosis score was positively related to the high infiltrated immune cells and negatively related to low infiltrated immune cells (CD4 memory resting T cells: R = 0.1081, P = 0.0157, follicular helper T cells: R = 0.1001, P = 0.0253, eosinophils: R = 0.1677, P = 0.0002, monocytes: R = 0.1492, P = 0.0008, M1 macrophages: R = 0.1034, P = 0.0209, M2 macrophages: R = -0.1919, P < 0.0001) (**Figures 6C–H**). We calculated the immune and stromal scores of thyroid cancer patients in TCGA and found that stromal score was increased in high fibrosis group and positively correlated with the fibrosis score, but no significant difference of immune score was detected between high and low fibrosis score groups (**Figures 6I, J**). Tertiary lymphoid structures (TLS) are the lymphoid tissue harboring architecture that highly associated with adaptive immune response (25). Recent studies reported that the presence of TLS indicated favorable outcomes in multiple tumors (25–27). According to the expression levels of 12 chemokines, we calculated TLS score of each TCGA-THCA cases. No significant difference of TLS score was displayed between high and low fibrosis groups (**Figure 6K**).

### Expression and survival analysis of 6 key fibrosis factors in thyroid cancer

**Figure 7:**
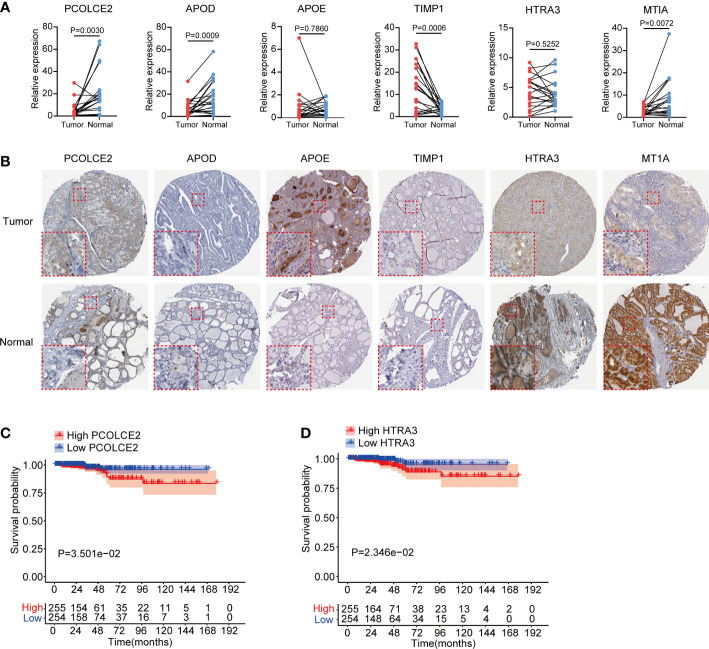
Expression validation and survival analysis of 6 key fibroblast related genes in thyroid cancer. **(A)** Transcriptional expression of PCOLCE2, APOD, APOE, TIMP1, HTRA3 and MT1A in 20 pairs clinical PTC and normal thyroid tissues. **(B)** Protein expression of PCOLCE2, APOD, APOE, TIMP1, HTRA3 and MT1A in normal and tumor thyroid tissues from Human Protein Atlas. **(C, D)** Kaplan–Meier plots showed that high expression of PCOLCE2 and HTRA3 were associated with poor overall survival probability for thyroid cancer patients.

We validated the transcriptional expression of identified 6 key fibrosis factors (PCOLCE2, APOD, APOE, TIMP1, HTRA3 and MT1A) in tumor and normal tissues obtained from 20 PTC patients (**Table S3**). Results showed that the expression of PCOLCE2, APOD and MTIA were increased, TIMP1 were down-regulated in tumor tissues, and no significant differences of APOE and HTRA3 were found between tumor and normal tissues (**Figure 7A**). Moreover, in an independent sequencing dataset, GSE33630 (23), including 49 PTC and 45 normal tissues, we found that PCOLCE2, APOD and HTRA3 were highly expressed in tumor tissues, the expression of APOE and TIMP1 were down-regulated (**Figure S4B**). The expression of MT1A was not found in GSE33630 dataset. The protein levels of 6 key fibrosis factors were explored in thyroid tumor and normal thyroid tissues using The Human Protein Atlas (HPA). Immunohistochemical indicated that APOE stained highly in thyroid tumor, while HTRA3 and MT1A showed the reverse staining results (**Figure 7B**). The protein expression of PCOLCE2, APOD and TIMP1 displayed no significant changes in tumor and normal thyroid tissues (**Figure 7B**). Kaplan–Meier plots suggested that high expression of PCOLCE2 and HTRA3 were associated with poor prognostic outcomes of thyroid cancer patients in TCGA (**Figures 7C, D**). However, no significant correlation was found between the expression of APOD, APOE, MT1A, TIMP1 and patients overall survival status (**Figures S4C–F**).

## Discussion

Fibroblasts are one of major stromal cells in the microenvironment of various tumors and have been proven to be critical in tumor development, including cell proliferation, immunosuppression, extracellular matrix remodeling and chemotherapy resistance (28–30). There are abundant of evidences indicating that highly infiltrated fibroblasts are associated with poor survival outcomes in various solid tumors (31–33). Previous research found that cancer-associated fibroblasts (CAFs) were highly enriched in dedifferentiated thyroid cancer (DDTC) and correlated with the aggressive outcomes of thyroid cancer patients (20). Pu, et al. identified two main fibroblast subpopulations in thyroid cancer, myofibroblastic CAFs (myoCAF) and inflammatory subtype (iCAF). Meanwhile, iCAF could interact with other type of cells in tumor environment (TME). Therefore, analyzing fibroblasts and exploring its molecular feature might provide a promising therapeutic target for thyroid cancer.

Single-cell RNA sequencing (scRNA-seq) technology has provided a powerful strategy to explore the intratumor heterogeneity and reveal the complex mechanisms in tumor environment (22, 34). In this study, we combined the scRNA and bulk RNA sequencing data of thyroid cancer and constructed a fibrosis related model to predict the prognosis for thyroid cancer patients. By analyzing the scRNA-seq data of 3 tumor and 3 normal thyroid tissues, we identified 11 cell types in the ecosystems of thyroid. For immune cells, we found CD4 T cells, B cells and plasma cells were low infiltrated in tumor tissues, while the infiltration levels of Treg, monocytes and myeloid cells were increased, indicating an immunosuppressive phenotype. Fibroblasts were highly infiltrated in tumor tissues, suggesting its critical function in thyroid cancer development. We explored the cell-cell interaction in thyroid tumor environment and found that fibroblasts could widely interact with other immune cells *via* Macrophage Migratory Inhibition Factor (MIF) signaling pathway. Previous studies reported that MIF was a multifunctional cytokine and inhibited immune functions in TME (35, 36). MIF interacted with CD74 to promote M2 immunosuppressive shift and inhibit M1 polarization, resulting in glioma development (37). In multiple myeloma patients, MIF promoted bone marrow stromal cells to secret the cytokines IL-6 and IL-8, which associated with poor prognosis (38). CXCL12−CXCR4 complex was another significant signaling pathway identified in cell-cell communication. As a receptor of CXCL12, CXCR4 was widely expressed in multiple cell types, such as lymphocytes, hematopoietic stem cells, endothelial cells, and malignant cells. The interacting of CXCL12 and CXCR4 could activate divergent intracellular pathways related to chemotaxis, cell proliferation and gene transcription in tumor development (39, 40). Yu et al. reported that CXCL12−CXCR4 activation promoted myeloid-derived suppressor cells (MDSCs) and macrophages infiltrations and accelerated colorectal progression (41). CXCL12−CXCR4 pathway antagonists combining immunotherapy had shown improving antitumor effects in HCC models (42). These evidences suggested that fibroblasts could regulate immune cell functions *via* cell-cell interactions in TME.

To investigate the molecular features of fibroblasts in thyroid cancer, we identified the gene list of fibroblasts cluster in sc-RNA seq data and analyzed their expression levels in TCGA-THCA dataset. Several fibroblasts related genes were differentially expressed between tumor and normal thyroid tissues, and associated with clinical stages of thyroid cancer patients. LASSO regression analysis was commonly applied to assess the transcriptome data and identify the most relevant factors associated with survival status of patients for risk model construction (43, 44). Here, we performed LASSO analysis and identified 6 critical fibroblasts related factors (PCOLCE2, APOD, APOE, TIMP1, HTRA3 and MT1A) and calculated the fibrosis scores based on their expression in patients with thyroid cancer. High fibrosis scores were associated with relatively short survival time and predicted optimal prognostic outcomes for patients in TCGA-THCA. As the most common genetic alteration in thyroid cancer, BRAF-V600E mutation existed in 57% patients from TCGA-THCA dataset (45, 46). Early researches reported BRAF mutation was associated with papillary thyroid carcinoma (PTC) long-term recurrence, metastasis, and advanced clinical stage (45–47). In south-east Asian thyroid cancer patients, no significant correlation was found between BRAF mutation and prognostic outcomes (46). Yang et al. found that CAFs infiltration was increased in thyroid cancer patients with BRAF-V600E mutation (48). However, in our study, the fibrosis score showed no significant difference between BRAF-V600E mutation and non-mutation groups. The DEGs between high and low fibrosis score groups in thyroid cancer were further identified. Functional enrichment analysis suggested that these DEGs might participate in various biological processes in thyroid cancer, such as endocrine system development, enzyme inhibitor activity and receptor binding. KEGG enrichment analysis identified several critical signal pathways including IL−17 signaling pathway, thyroid hormone signaling pathway and transcriptional mis-regulation in cancer.

Thyroid cancer development was often associated with chronic inflammation, which indicated that the immune cells were critical components in TME and played critical roles in cytokines secretion to maintain immune response (49). Thyroid cancer cells could suppress the cytolytic function of NK cells by secreting prostaglandin E2 and cyclooxygenase-2 to promote tumor progression (50). By bioinformatic analysis, early researches studied the TME changes and found that several types of immune cell were dis-regulated in thyroid cancer, such as CD8 T cells, macrophages, Tregs, monocytes and neutrophils (13, 48). Moreover, researchers demonstrated that the tertiary lymphoid structures (TLS) scores were decreased in thyroid cancer along with down-regulated chemokines (48). As an ectopic lymphoid structures, TLS was a T cell zone consisted of T cells, B cells, follicular dendritic cells, neutrophils, and so on and act as antitumor roles in adaptive immune response (51). The presence of TLS was often correlated with beneficial clinical outcomes in patients with cancer (51). To evaluate the association between immune cells and fibrosis score, we compared the infiltration levels of immune cells between high and low fibrosis score thyroid cancer patients. We found that fibrosis score was positively related to monocytes, M1 macrophages and eosinophils infiltration, but negatively correlated with M2 macrophages. Meanwhile, the stromal score rather than immune score or TLS score of thyroid cancer patients was increased in high fibrosis score group patients due to fibroblasts belonging to stromal cells in TME.

The expression levels of 6 critical fibroblasts-related factors were validated in 20 pairs clinical PTC tissues and another independent cohort. The protein levels of HTRA3 and MT1A were significantly attenuated, but APOE protein level was increased in thyroid tumor. Survival analysis suggested that high expression level of PCOLCE2 and HTRA3 were related to poor overall survival results for thyroid cancer patients. HTRA3 was a serine peptidase and had been reported to participate in multiple signal pathways in malignancies. High expression of HTRA3 was associated with advanced clinical stage and indicated poor overall survival proportion in gastric cancer (52). In colorectal cancer, researchers identified that HTRA3 could be expressed by tumor cells and peritumoral stromal cells (53). MT1A was a member of metallothioneins and played a critical role in metal homeostasis and oxidative stress (54). However, the expression and function of MT1A in tumor progression remains controversial. It has been reported that MT1A was highly expressed and associated with shorter survival time in astrocytoma and lung cancer patients (55, 56). But in oral squamous cell carcinoma, MTIA expression was significantly decreased (57). PCOLCE2 was reported to mainly expressed in heart and participated in procollagen processing and fibrillar collagen deposition (58). Besides, PCOLCE2 was identified as a key factor in tumor epithelial-mesenchymal transition (EMT) and ferroptosis (59, 60). Here, we showed that PCOLCE2 and HTRA3 were mainly expressed by fibroblasts in thyroid cancer and decreased in tumor tissues. However, low expression of PCOLCE2 and HTRA3 suggested favorable clinical outcomes for thyroid cancer patients. There are still some limitations in our study. Firstly, we constructed the fibrosis score model for thyroid cancer patients only from TCGA cohort due to the difficulty to obtain public datasets containing both transcriptomic and survival data, which might lead to selection bias. Therefore, large numbers of clinical samples are required to evaluate the clinical applicability of fibrosis score model. Secondly, the expression of PCOLCE2 and HTRA3 were decreased in thyroid tumor and indicated favorable prognosis for thyroid cancer patients, which is contradictory to the conventional perception. Insights into the molecular mechanisms of PCOLCE2 and HTRA3 need to be further elucidated by experimental researches.

## Conclusions

In conclusion, our study revealed the expression profile and prognostic values of fibroblasts in thyroid cancer combining single-cell and bulk RNA sequencing data. We constructed a novel fibrosis score model including 6 key fibroblasts related factors (PCOLCE2, APOD, APOE, TIMP1, HTRA3 and MT1A). High fibrosis score is characterized with specific immune cells infiltration and leads to poor clinical survival for thyroid cancer patients. Our research may provide novel horizons about fibroblasts and potential therapeutic targets for PTC patients.

## Data availability statement

The datasets presented in this study can be found in online repositories. The names of the repository/repositories and accession number(s) can be found in the article/**Supplementary Material**.

## Ethics statement

The studies involving human participants were reviewed and approved by Research and Ethics Committee of Changzheng Hospital. The patients/participants provided their written informed consent to participate in this study.

## Author contributions

WL, ZL, XC, and MQ generated the hypothesis and designed the study. WL and ZL collected the data. WL, ZL, XC, JX, SZ, BW, and WZ analyzed and interpreted the data. WL, ZL, XC, BW, WZ, and MQ wrote the manuscript. The authors read and approved the final manuscript.

## Funding

This research is supported by the Youth Foundation of Shanghai Changzheng Hospital (type A, 2020-2023).

## Conflict of interest

The authors declare that the research was conducted in the absence of any commercial or financial relationships that could be construed as a potential conflict of interest.

## Publisher’s note

All claims expressed in this article are solely those of the authors and do not necessarily represent those of their affiliated organizations, or those of the publisher, the editors and the reviewers. Any product that may be evaluated in this article, or claim that may be made by its manufacturer, is not guaranteed or endorsed by the publisher.

## Supplementary material

Supplementary Figure 1 The dot plot of cell markers and cell-cell communications in thyroid cancer. **(A)** The dot plot of cell markers for different cell clusters. **(B)** Proportions of different cell clusters in each sample tissue. **(C)** Integrated cell-cell communications network plotted by interaction numbers. **(D)** Cell-cell communications network of each cell cluster.

Supplementary Figure 2 Cell-cell communications heatmap in thyroid cancer. **(A)** The heatmap of incoming and outgoing signaling patterns. **(B)** The heatmap of MIF signaling pathway network.

Supplementary Figure 3 Expression heatmap of fibroblast related genes and fibrosis scores in TCGA-THCA dataset. **(A)** The expression heatmap of FRGs between normal and tumor thyroid cancer. **(B)** The samples in TCGA-THCA dataset were divided into high and low fibrosis groups based on the median score.

Supplementary Figure 4 Immune cells infiltration and 6 key genes analysis in thyroid cancer patients. **(A)** Immune cells infiltration proportions in each thyroid cancer patients. **(B)** Expression levels of PCOLCE2, APOD, APOE, TIMP1 and HTRA3 in GSE33630. **(C–F)** The expression of MT1A was not found in this cohort. Kaplan–Meier plots showed the expression of APOD, APOE, MT1A and TIMP1 were not associated with overall survival of thyroid cancer patients.
