# Supplementary figures and images for: Integrated analysis of fibroblasts molecular features in papillary thyroid cancer combining single-cell and bulk RNA sequencing technology
Source: Front Endocrinol (Lausanne). 2022 Oct 26;13:1019072. doi: 10.3389/fendo.2022.1019072 (PMC9643292; doi:10.3389/fendo.2022.1019072)

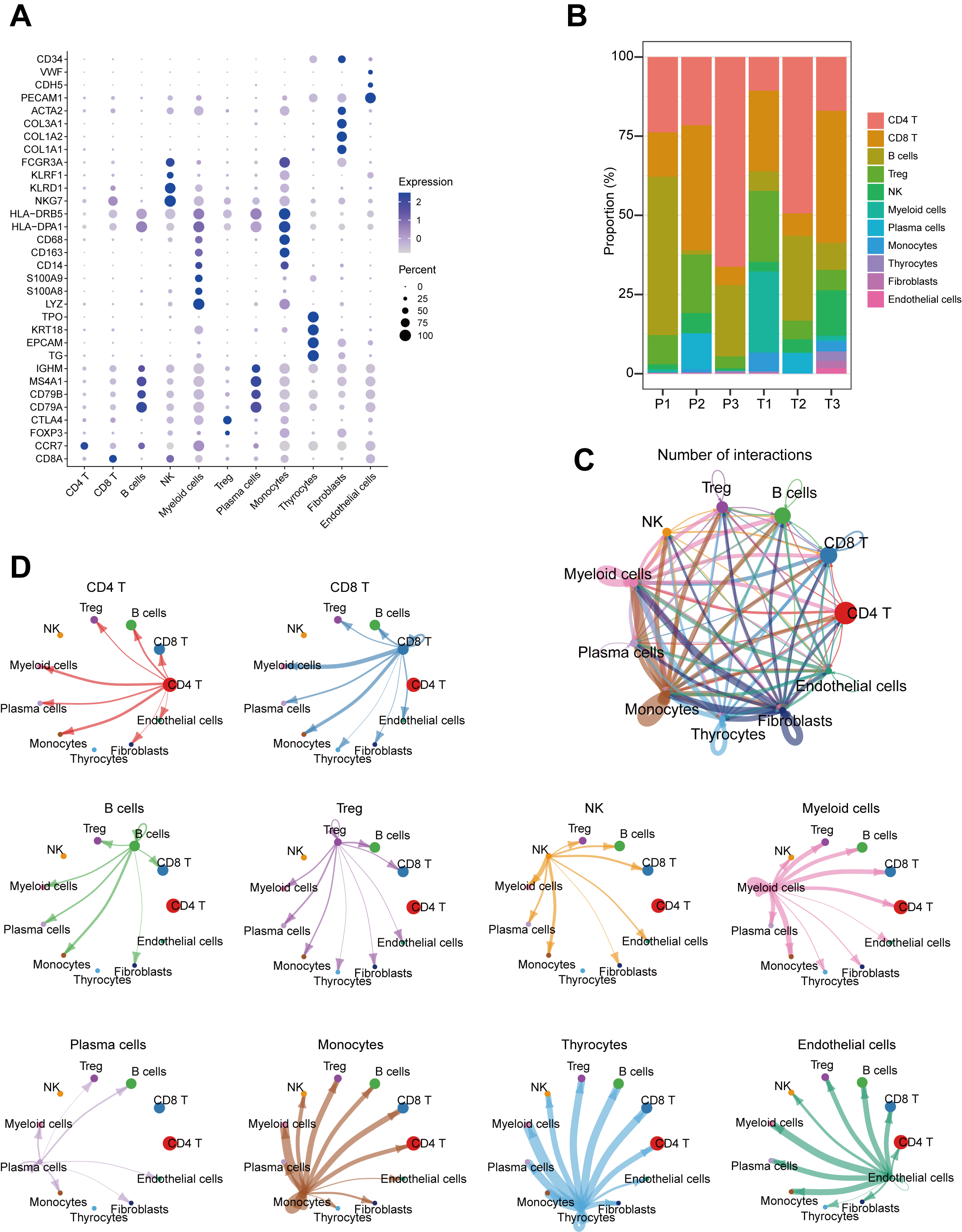

Supplement: Supplementary Figure 1 — The dot plot of cell markers and cell-cell communications in thyroid cancer. (A) The dot plot of cell markers for different cell clusters. (B) Proportions of different cell clusters in each sample tissue. (C) Integrated cell-cell communications network plotted by interaction numbers. (D) Cell-cell communications network of each cell cluster. [file Image_1.tif]

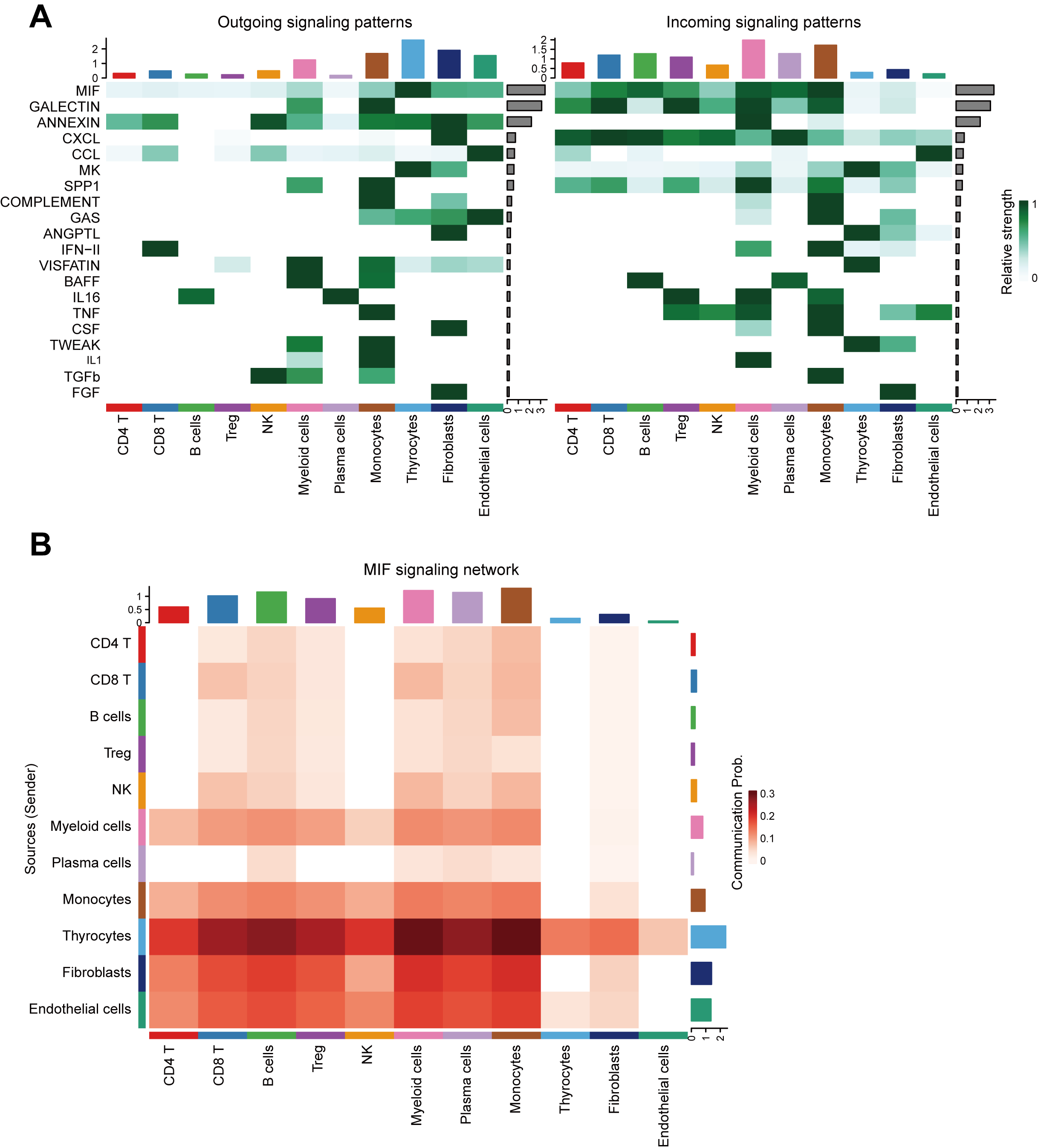

Supplement: Supplementary Figure 2 — Cell-cell communications heatmap in thyroid cancer. (A) The heatmap of incoming and outgoing signaling patterns. (B) The heatmap of MIF signaling pathway network. [file Image_2.tif]

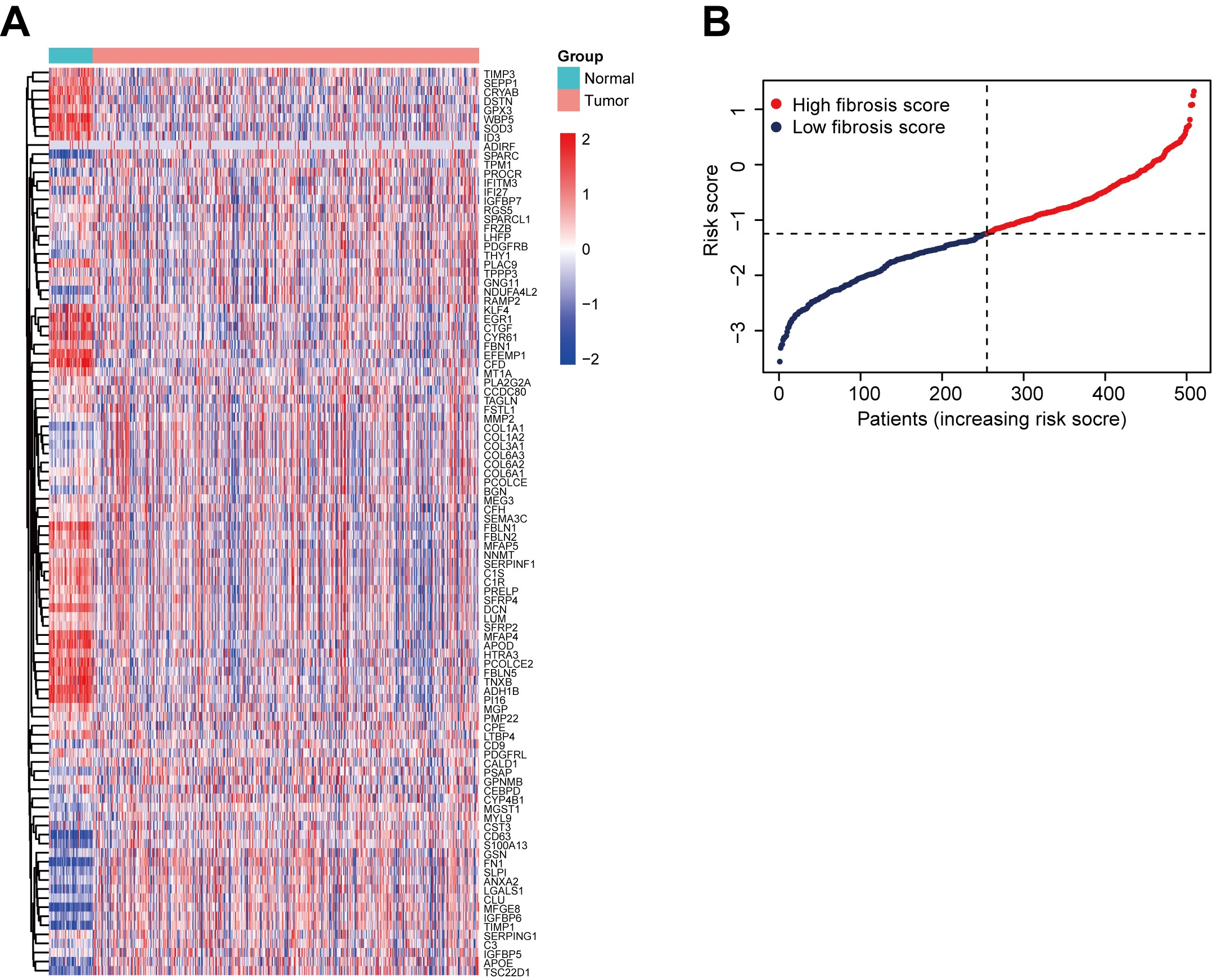

Supplement: Supplementary Figure 3 — Expression heatmap of fibroblast related genes and fibrosis scores in TCGA-THCA dataset. (A) The expression heatmap of FRGs between normal and tumor thyroid cancer. (B) The samples in TCGA-THCA dataset were divided into high and low fibrosis groups based on the median score. [file Image_3.tif]

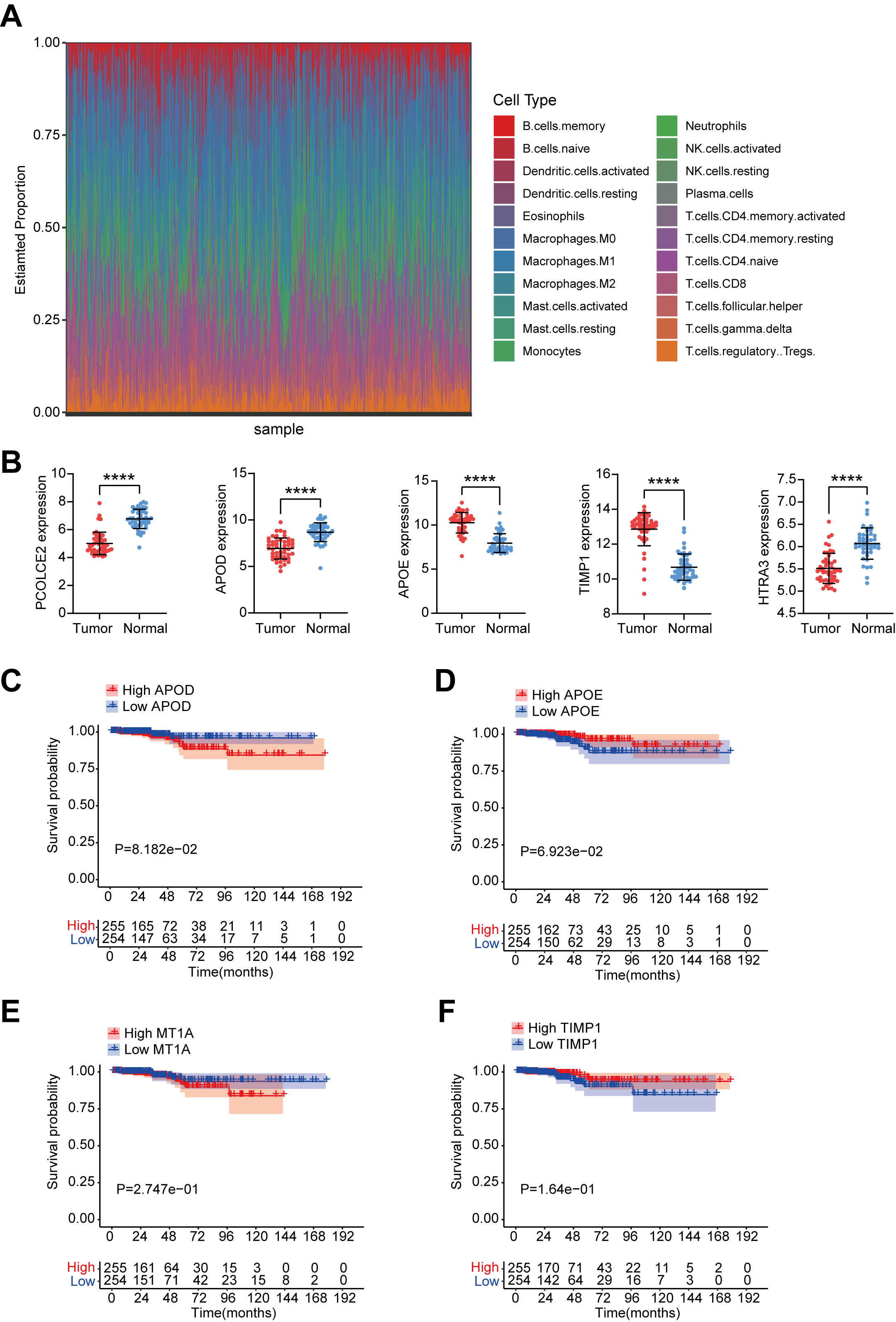

Supplement: Supplementary Figure 4 — Immune cells infiltration and 6 key genes analysis in thyroid cancer patients. (A) Immune cells infiltration proportions in each thyroid cancer patients. (B) Expression levels of PCOLCE2, APOD, APOE, TIMP1 and HTRA3 in GSE33630. (C–F) The expression of MT1A was not found in this cohort. Kaplan–Meier plots showed the expression of APOD, APOE, MT1A and TIMP1 were not associated with overall survival of thyroid cancer patients. [file Image_4.tif]
