# Supplementary material for: Integrated analysis of fibroblasts molecular features in papillary thyroid cancer combining single-cell and bulk RNA sequencing technology
Source: Front Endocrinol (Lausanne). 2022 Oct 26;13:1019072. doi: 10.3389/fendo.2022.1019072 (PMC9643292; doi:10.3389/fendo.2022.1019072)
Supplement: Supplementary file 7 [file Table_3.docx]

Supplementary Table 3：Clinical characteristics of 20 PTC patients.

| Clinicopathological parameters | Frequency | Percentage |
| --- | --- | --- |
| Gender | | |
| Male | 5 | 25.0% |
| Female | 15 | 75.0% |
| Age | | |
| <50 | 14 | 70% |
| ≥50 | 6 | 30% |
| BMI | | |
| <24 | 8 | 40% |
| ≥24 | 12 | 60% |
| T3 (nmol/L) | 1.65 ± 0.22 | - |
| T4 (nmol/L) | 82.98 ± 12.53 | - |
| FT3 (pmol/L) | 4.62 ± 0.56 | - |
| FT4 (pmol/L) | 14.65 ± 2.59 | - |
| TSH (mU/L) | 2.36 ± 1.29 | - |
| TG-Ab | | |
| Negative | 16 | 80% |
| Positive | 4 | 20% |
| TPO-Ab | | |
| Negative | 17 | 85% |
| Positive | 3 | 15% |
| Hashimoto thyroiditis | | |
| Negative | 15 | 75% |
| Positive | 5 | 25% |
| The largest diameter of tumor (cm) | 0.83 ± 0.51 | - |
| Tumor numbers | | |
| 1 | 14 | 70% |
| >1 | 6 | 30% |
